# Panhypopituitarism: A Case Report of a Rare Complication of Cerebral Venous Thrombosis Secondary to Ulcerative Colitis

**DOI:** 10.7759/cureus.117868

**Published:** 2026-10-13

**Authors:** Pyaesone Nyunt, Aye Nyunt

**Affiliations:** 1 Diabetes and Endocrinology, Barts Health NHS Trust, London, GBR; 2 Diabetes and Endocrinology, Glan Clwyd Hospital, Rhyl, GBR

**Keywords:** cerebral venous sinus thrombosis (cvst), empty sella, inflammatory bowel disease, panhypopituitarism, ulcerative colıtıs

## Abstract

Cerebral venous sinus thrombosis (CVST) is a recognised but uncommon thrombotic complication of ulcerative colitis (UC). Hypothalamic-pituitary dysfunction, specifically panhypopituitarism following extensive cerebral venous thrombosis, has been rarely documented. We report a case of a 21-year-old gentleman with a background of recently diagnosed UC. On his first admission, he developed symptoms suggestive of CVST, which required intensive therapy unit (ITU) admission and was later discharged. Four months later, he was re-admitted due to a severe UC flare resulting in toxic megacolon, requiring a subtotal colectomy and further ITU admission. During the course of his admission, he developed polyuria, hypernatraemia, raised plasma osmolality, recurrent hypoglycaemia, and hypogonadism. Upon endocrine consultation, the assessment concluded that there was evidence of anterior and posterior pituitary dysfunction in keeping with panhypopituitarism. He was treated with hydrocortisone, levothyroxine, desmopressin, and testosterone replacement. A follow-up pituitary MRI later showed appearances consistent with empty sella. Overall, this case highlights two important considerations. Firstly, early recognition of CVST is needed in patients with neurological symptoms and active UC. Secondly, pituitary dysfunction should be considered in patients with UC who develop extensive CVST alongside persistently unexplained hypoglycaemia, polyuria, hypernatraemia, or hyperosmolality.

## Introduction

Ulcerative colitis (UC) is a form of chronic inflammatory bowel disease (IBD), characterised by continuous mucosal inflammation that traditionally develops from the rectum and can extend to the entire colon. UC is also associated with extra-intestinal features, most commonly anaemia, peri-articular or axial arthropathy, and ocular and cutaneous manifestations [1]. The most relevant extra-intestinal complication in this case report is the risk of venous thromboembolism (VTE), particularly during inflammation and physical stress [2]. It is recognised that those with IBD have an up to fourfold increased risk of developing VTE, compared with the general population [3]. Common thromboembolic events include pulmonary emboli (PE) and deep vein thrombosis (DVT), although our main focus is on the development of cerebral venous sinus thrombosis (CVST) [2].

Following the development of CVST, there is a further risk of endocrine pathology manifesting, specifically, hypopituitarism. This form of pituitary dysfunction involves either complete or partial impairment of the anterior or posterior pituitary hormones, with complete deficiency referred to as panhypopituitarism [4]. Hypopituitarism itself is known to be uncommon and has a history of being underdiagnosed, which further highlights the importance of recognising its features [4]. This unusual case report will detail this young gentleman's journey, outlining his initial clinical presentation, leading to the diagnosis of CVST and panhypopituitarism, as well as the management and follow-up thereafter.

## Case presentation

A 21-year-old man with a three-month history of UC presented to the emergency department with a 12-day history of progressive headache, dizziness, blurred vision, and photophobia. Later that day, he developed agitation, confusion, and altered speech. A CT brain scan showed increased density within the cerebral venous sinuses, suggestive of thrombosis (Figure 1).

**Figure 1:**
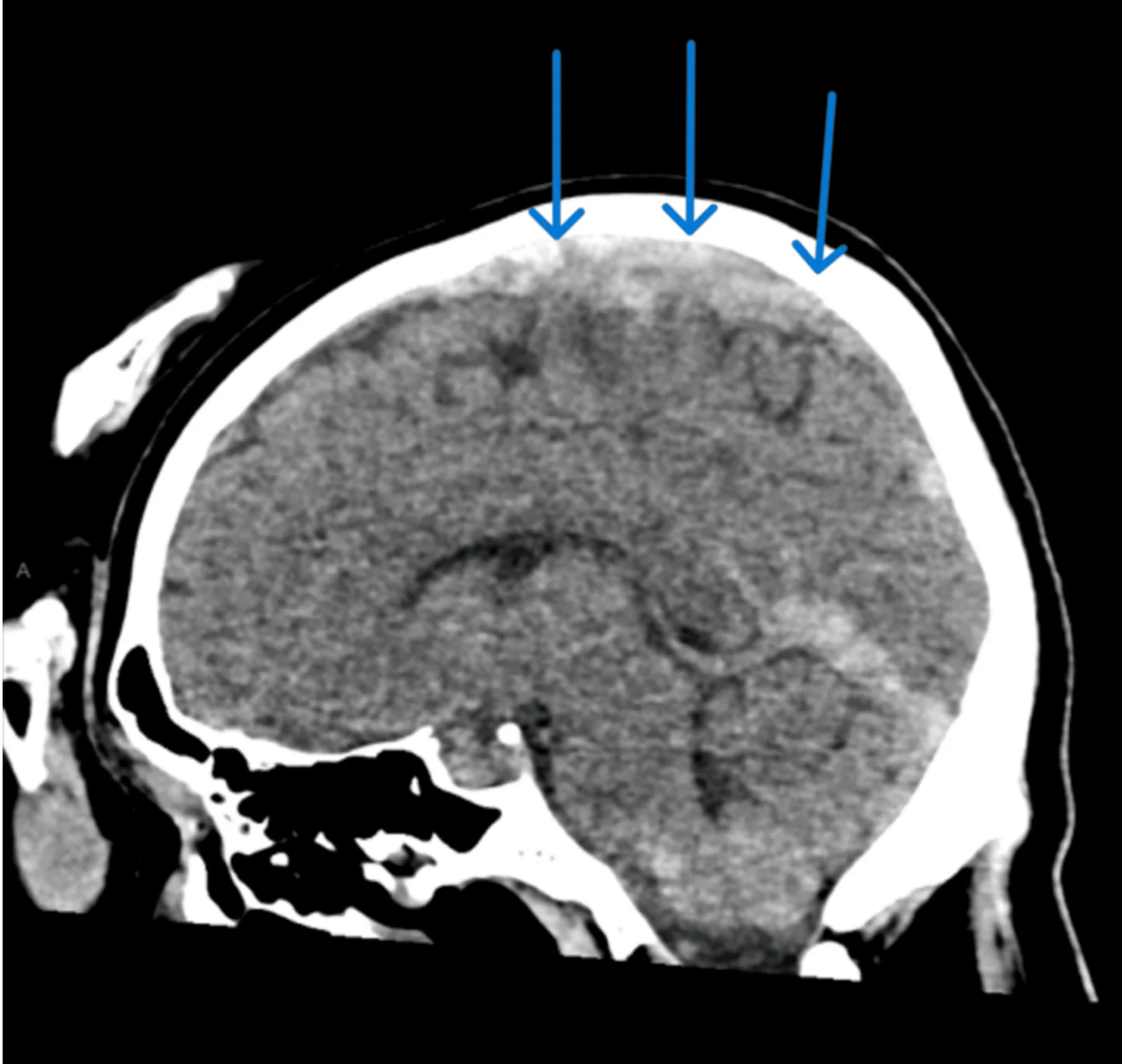
Sagittal view of a non-contrast CT head showing extensive high density in the cerebral venous sinuses (blue arrows).

CT venography confirmed extensive occlusive filling defects involving most of the superior sagittal sinus, the straight sinus, the right transverse and sigmoid sinuses, and the right proximal internal jugular vein, with partial filling defects in the left transverse and sigmoid sinuses and the left proximal internal jugular vein (Figure 2). 

**Figure 2:**
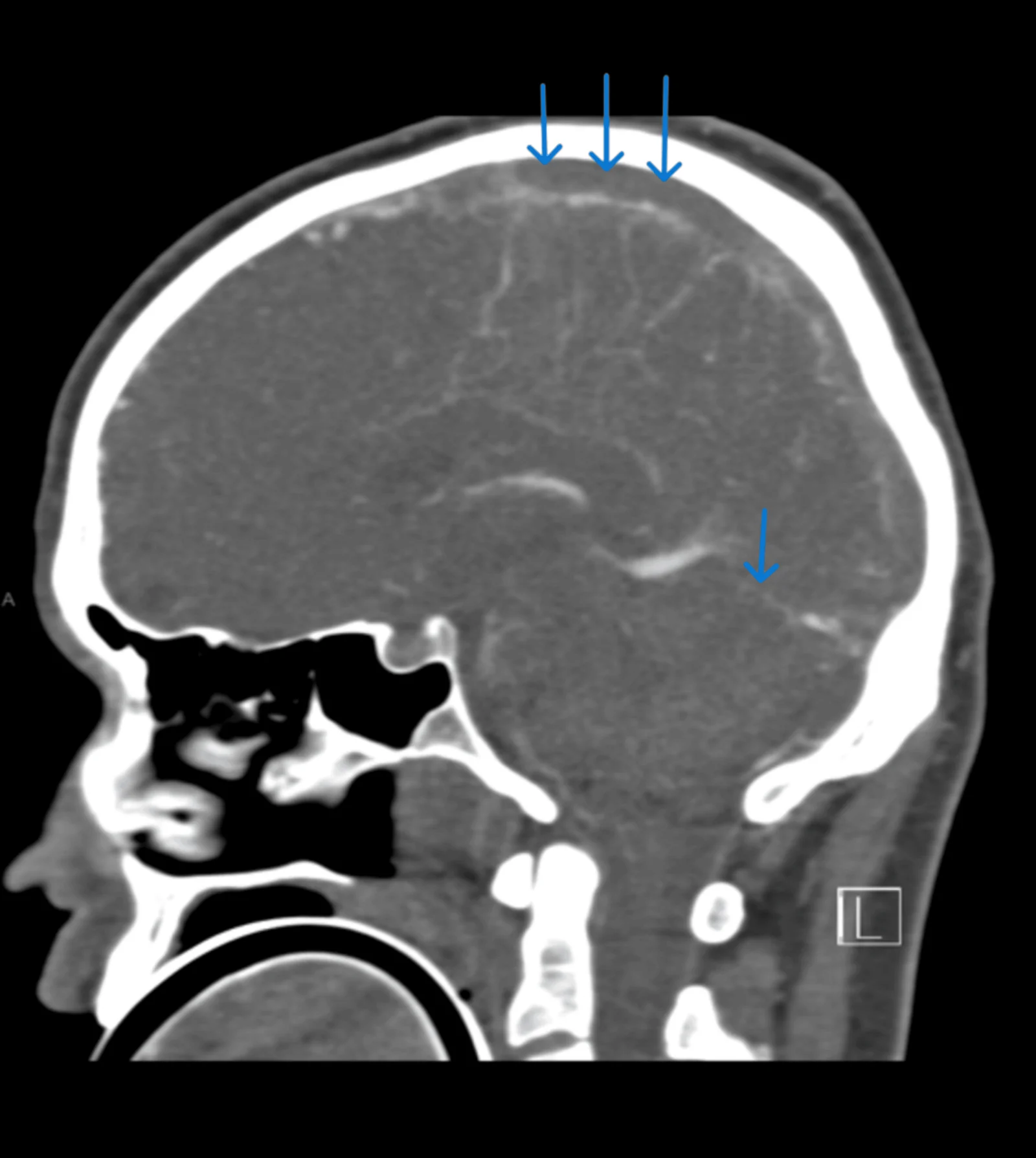
Sagittal view of a CT venogram showing the absence of contrast filling within the sagittal and transverse sinuses, consistent with cerebral venous thrombosis (blue arrows).

He was then intubated and ventilated and admitted to ITU; his pre-intubation Glasgow Coma Scale score was 9 (E3 V2 M4) [5]. He was treated with intravenous (IV) heparin followed by split-dose low-molecular-weight heparin (LMWH). Blood cultures, cerebrospinal fluid analysis and thrombophilia screening were negative. Ophthalmology review identified stable grade 4 papilloedema, and acetazolamide was commenced. He was later discharged home on LMWH with follow-up by the haematology and gastroenterology teams.

Two months later, the patient had a subsequent admission due to a UC flare, presenting with reduced appetite, dysphagia, vomiting and bloody diarrhoea. His UC initially responded well to infliximab; however, treatment was complicated by acute hepatitis, and therapy was changed to adalimumab, as well as a weaning dose of oral prednisolone (initially IV hydrocortisone for seven days). He was also treated for oral candidiasis, which resolved with oral fluconazole. The gentleman continued on anticoagulation for CVST and continued to be followed up by the gastroenterology, ophthalmology and haematology teams.

Four months after the CVST, he re-presented to the emergency department, with ongoing symptoms of a UC flare. He subsequently developed a left leg DVT while receiving enoxaparin, needing an inferior vena cava filter insertion. Despite treatment with adalimumab, he continued to have bloody diarrhoea several times per day. He underwent emergency laparotomy and subtotal colectomy with ileostomy after perforation of the transverse colon secondary to toxic megacolon and was managed in ITU.

Three days post-operatively, while in intensive care, he developed polyuria, with a urine output of 3.5-4.8 L per day, associated with a rising serum sodium of 151-159 mmol/L and serum plasma osmolality of 316-342 mOsm/kg. Despite parenteral and nasogastric feeding, he had recurrent hypoglycaemia, with glucose levels of 2.9-3.1 mmol/L occurring four to six times per day. Endocrinology review identified biochemical evidence of pituitary dysfunction: adrenocorticotropic hormone ACTH <1.5 ng/L, peak cortisol 327 nmol/L, insulin-like growth factor 1 (IGF-1) 14.9 nmol/L, testosterone 0.4 nmol/L, follicle-stimulating hormone (FSH) 3.6 IU/L, luteinising hormone (LH) 5.0 IU/L, free T4 9.9 pmol/L, thyroid-stimulating hormone (TSH) 1.61 mU/L, and prolactin 497 mIU/L (Table 1). The clinical picture was consistent with panhypopituitarism, with features suggestive of both anterior and posterior pituitary involvement. MRI of the pituitary and brain showed no focal pituitary lesion, no acute infarction, and no space-occupying lesion (Figure 3). He was commenced on IV hydrocortisone, levothyroxine, and desmopressin, followed by testosterone replacement therapy.

**Table 1:** Biochemistry table of results outlining reduced levels of ACTH, cortisol, testosterone, free T4, and serum glucose, alongside evidence of raised serum sodium and serum osmolality, suggesting the presence of panhypopituitarism. ACTH: adrenocorticotropic hormone; FSH: follicle-stimulating hormone; LH: luteinising hormone; IGF-1: insulin-like growth factor 1; TSH: thyroid-stimulating hormone.

Hormone/electrolytes	Results	Reference ranges
ACTH	<1.5 ng/L	7-63 ng/L
Cortisol (0 min)	203 nmol/L	>420 nmol/L
30-Min peak cortisol (short Synacthen test)	327 nmol/L	>420 nmol/L
Testosterone	0.4 nmol/L	9-31 nmol/L
FSH	3.6 IU/L	1.5-12.4 IU/L
LH	5 IU/L	1.7-8.6 IU/L
Prolactin	497 mU/L	85-350 mU/L
IGF-1	14.9 nmol/L	15-46.1 nmol/L
Free T4	9.9 pmol/L	11-25 pmol/L
TSH	1.61 mU/L	0.27-4.2 mU/L
Serum sodium (Na)	151-159 mmol/L	133-146 mmol/L
Serum osmolality	316-342 mOsm/kg	275-295 mmol/kg
Serum glucose	2.9-3.1 mmol/L	3.9-5.5 mmol/L

**Figure 3:**
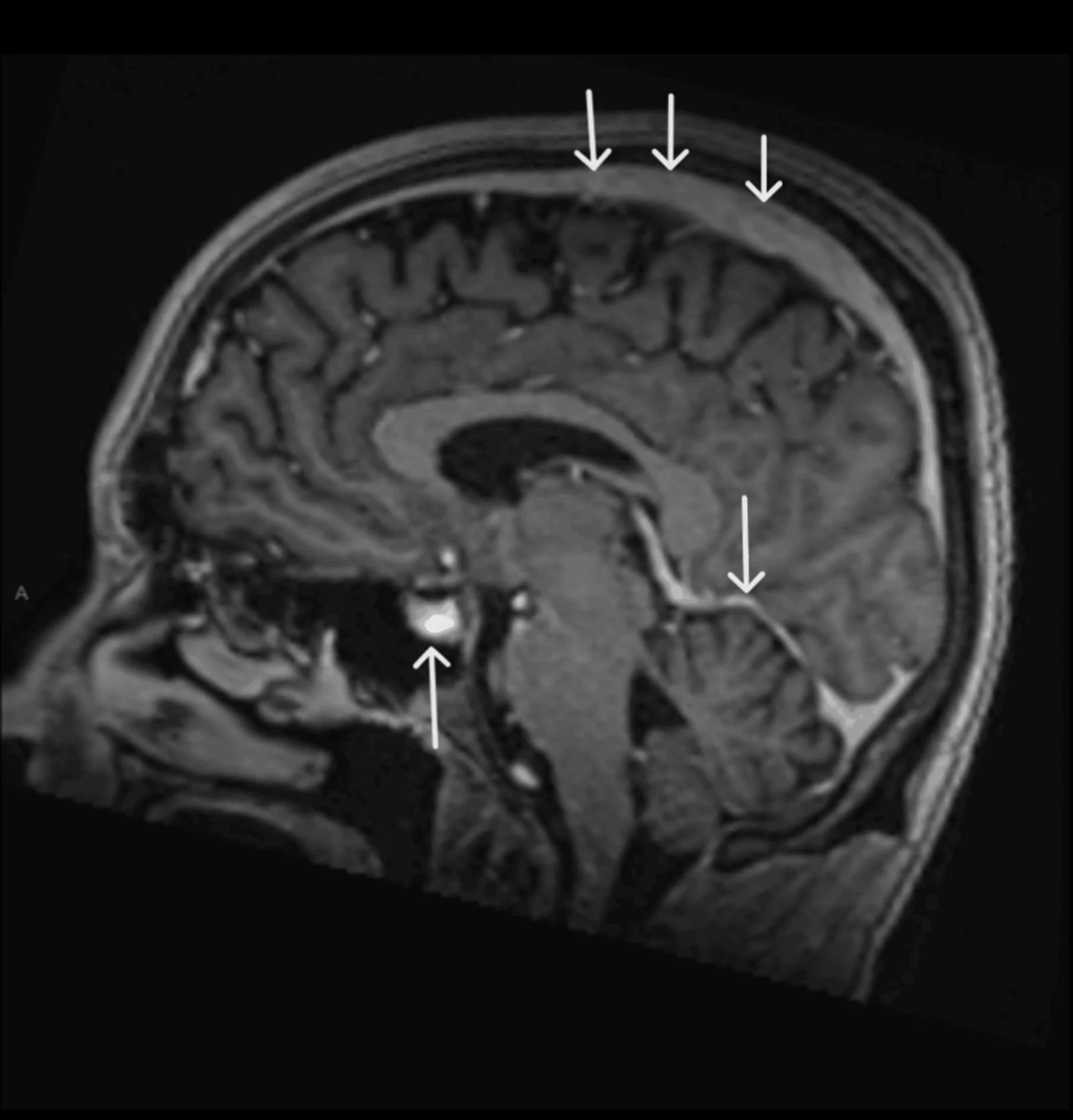
Sagittal section of pituitary MRI, T1-weighted sequence, during the acute admission, showing no focal pituitary lesion and now showing interval sagittal and transverse venous sinus contrast filling (white arrows).

He required a prolonged 100-day admission to the ITU. In addition to panhypopituitarism, his admission was complicated by hospital-acquired pneumonia, pyoderma gangrenosum affecting both legs, and abdominal wound dehiscence. He was discharged to a community hospital for further rehabilitation for three months and continued to be followed up by the surgical team, stoma nurse, tissue viability team, ophthalmology, and endocrinology teams. Eleven months after hospital discharge, repeat pituitary MRI showed a very thin pituitary gland confined to the floor of the sella turcica, with appearances consistent with empty sella (Figure 4).

**Figure 4:**
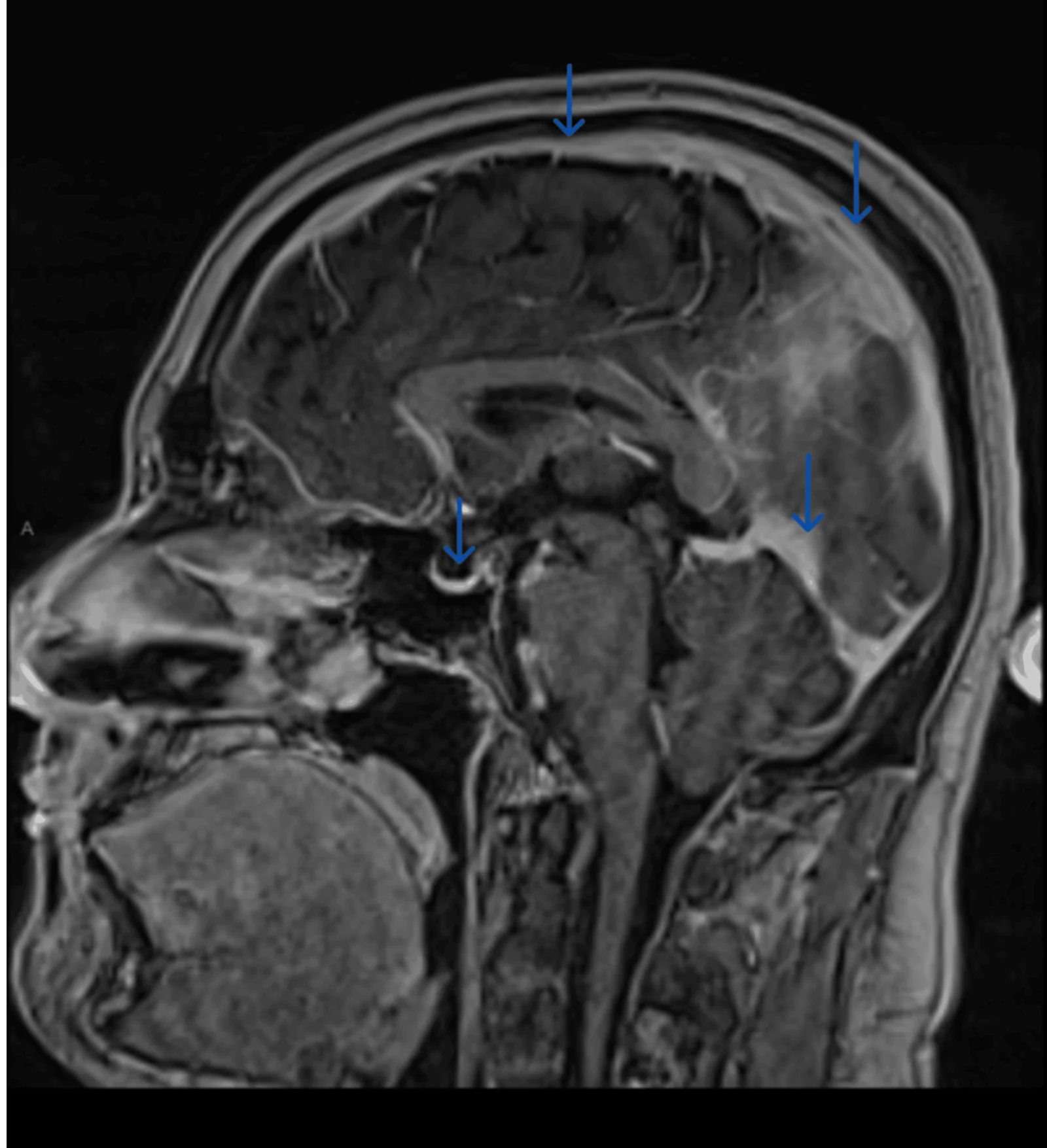
Follow-up sagittal section of pituitary MRI, T1-weighted sequence, showing a thin pituitary gland with patent venous sinuses, in keeping with empty sella (blue arrows).

## Discussion

CVST most commonly presents with headache, alongside possible features of visual changes, papilloedema, and altered mental status, which were seen in the clinical presentation of our patient; known risk factors, predominantly seen in younger women, include the use of oral contraceptive medication and oestrogen-based hormonal therapies [6]. However, the pathophysiology and predisposing factors of CVST associated with UC remain multifactorial and are not completely understood. It is theorised that active inflammation during a UC flare may promote a prothrombotic state, leading to an increased VTE risk due to reduced protein C, protein S, and antithrombin levels, alongside increased fibrinogen and factor VIII levels [2,7]. Although its incidence within a cohort of IBD patients is uncommon at 0.48%, as demonstrated in one retrospective study, as well as the incidence of four cases over a span of 10 years documented in a series of case reports, CVST remains a life-threatening extra-intestinal manifestation of UC if not managed promptly [3,8,9].

Reports of hypopituitarism as a complication secondary to CVST are limited, with an example of seven known cases that occurred due to cavernous sinus thrombosis (CST), a specific form of CVST [10]. To give context to these cases, formation was secondary to infection, with an emphasis on initial surgical drainage and antibiotic therapy as management, as opposed to solely a focus on anticoagulation as the mainstay of treatment for aseptic CVST [6,10]. In terms of how anterior hypopituitarism developed in these cases, a theorised mechanism is the reduction in venous circulation, with specific impairment of hypophyseal vessels in relation to their supply to the anterior pituitary, leading to pituitary ischaemia or infarction [10,11]. Regarding posterior hypopituitarism, this is not as common due to the synthesis of posterior pituitary hormones in the hypothalamus; a previous report of panhypopituitarism secondary to septic CST hypothesises posterior pituitary dysfunction due to hypothalamic infarction, while involving inflammation of the carotid artery [10]. For our patient who developed panhypopituitarism secondary to CVST, there were extensive filling defects within the cerebral venous system with no prior septic features, nor evidence of carotid artery involvement. 

An important factor in the development of hypopituitarism secondary to CVST is the timing of when it occurs: it has been known to be present within the acute phase, meaning less than 12 months from symptom onset, as well as more than 12 months since the event [12]. There was one study in which a patient regained endocrine function and fully recovered, although before this there have been documented cases in which patients were left with ongoing pituitary dysfunction [12]. In our patient, panhypopituitarism has persisted despite treatment of the underlying thrombotic disease with anticoagulation. He has required ongoing hormone replacement therapy for three years without evidence of recovery of pituitary function, suggesting substantial or irreversible injury to the hypothalamic-pituitary axis.

This case report therefore highlights two important clinical considerations. Firstly, despite its rarity, CVST should be considered in patients with an active UC flare who develop new neurological symptoms, including early intervention to improve prognosis. Fortunately, there was full resolution of CVST despite poor prognostic factors such as impaired consciousness in our patient [6]. Secondly, clinicians should be made aware and be alerted to the possibility of hypothalamic-pituitary dysfunction in patients with CVST and familiarise themselves with features of endocrine failure such as unexplained electrolyte abnormalities, hypotension, fatigue, and impaired consciousness. Early recognition is important, given the increased rate of mortality in patients with hypopituitarism [4].

## Conclusions

This case illustrates a rare endocrine complication following extensive CVST in the setting of an active UC flare. Although CVST is a recognised thrombotic complication of IBD, pituitary dysfunction is rarely considered in this context. Early endocrine assessment should be considered when unexplained hypoglycaemia, polyuria, hypernatraemia, or hyperosmolality develops after CVST, particularly in critically ill patients in whom adrenal insufficiency, central hypothyroidism, and diabetes insipidus may be clinically masked; this should aim to reduce the morbidity and mortality rate of this patient group.

Persistent panhypopituitarism that has lasted three years in this patient also emphasises the importance of long-term endocrine follow-up. It is possible to recover pituitary function; however, the duration and extent of recovery are unpredictable. Therefore, ongoing assessments are required to determine whether hormone replacement is needed and for how long.

## References

[1] Segal JP, LeBlanc JF, Hart AL (2021). Ulcerative colitis: an update. Clin Med [Internet].

[2] Magro F, Soares JB, Fernandes D (2014). Venous thrombosis and prothrombotic factors in inflammatory bowel disease. World J Gastroenterol.

[3] Shujun W, Huijie Z, Xia B, Hongjian W (2021). Cerebral venous sinus thrombosis in patients with inflammatory bowel disease: a retrospective study. Sci Rep.

[4] Fleseriu M, Mirjam Christ-Crain, Langlois F, Mônica Gadelha, Melmed S (20241). Hypopituitarism. Lancet.

[5] Teasdale G, Jennett B (1974). Assessment of coma and impaired consciousness. A practical scale. Lancet.

[6] Saposnik G, Bushnell C, Coutinho JM (2024). Diagnosis and management of cerebral venous thrombosis: a scientific statement from the American Heart Association. Stroke.

[7] Hamid M, Ahizoune A, Berri MA (2023). Cerebral venous thrombosis secondary to ulcerative colitis: a case report with a literature review. Radiol Case Rep.

[8] Crook SM, Quinton RA (2021). Cerebral vascular thrombosis associated with ulcerative colitis and primary sclerosing cholangitis. Am J Forensic Med Pathol.

[9] Yehia A, Mudege T, Hoque S (2025). Cerebral vein sinus thrombosis, an unusual complication of inflammatory bowel disease. Eur J Case Rep Intern Med.

[10] Desa V, Green R (2012). Cavernous sinus thrombosis: current therapy. J Oral Maxillofac Surg.

[11] Petty RK, Wardlaw J, Kennedy PG, O'Reilly B, McFadzean R, Johnson RA, Davies DL (1994). Panhypopituitarism after cavernous sinus thrombosis. J Neurol Neurosurg Psychiatry.

[12] Hladky JP, Leys D, Vantyghem MC (1991). Early hypopituitarism following cavernous sinus thrombosis: total recovery within 1 year. Clinical Neurol Neurosurg.

